# The Search for an Ideal Wound Dressing

**Published:** 1984-07

**Authors:** Sean S. Brennan, David J. Leaper

**Affiliations:** University Department of Surgery, Bristol Royal Infirmary, Bristol BS2 8HW; University Department of Surgery, Bristol Royal Infirmary, Bristol BS2 8HW


### The Search for an Ideal Wound Dressing

**Sean S. Brennan, F.R.C.S.**
Research Fellow
**David J. Leaper, M.D., Ch.M., F.R.C.S.**
Consultant Senior Lecturer, University Department of Surgery, Bristol Royal Infirmary, Bristol BS2 8HW

The earliest record of wound management is written on a Sumerian clay tablet dating from about 2500 B.C. It describes the cleansing of wounds with water and milk followed by a dressing with honey and resin. The Egyptians discovered that a closed wound will heal faster than a wound which is left open. They devised an adhesive dressing by applying gum to linen strips which were applied to draw the edges of an open wound together. The Ebers papyrus advocates dressing open wounds with honey, or resins such as frankincense and myrrh. The Egyptians also used a green copper pigment obtained from malachite or chryscolla, which was also used as a colouring for eye adornment, and has since been shown to possess antiseptic properties.

Celsus is remembered in the history of medicine as the physician who first described the four cardinal signs of inflammation. He recognised that fresh wounds and open chronic sores required quite different management to achieve healing. Galen was another excellent clinical observer of the same era but where direct observation failed him he fell back on philosophical arguments to explain disease processes. Because of his doctrines progress in the management of wounds almost came to a standstill and indeed regressed for the following 1500 years. Noxious substances were placed in wounds in order to encourage 'pus bonum et laudabile' which was considered a necessity prior to successful healing. Faeces, cobwebs, boiling oil and cautery became accepted forms of wound management during the Middle Ages. Certain faults of the Galenic doctrines were recognised by a few enlightened practitioners such as Ambroise Pare but it was only after the Renaissance that advances were made in the understanding of wound sepsis.

In the late eighteenth and early nineteenth centuries advances in chemistry made elemental iodine and chlorine available and in 1846 Semmelweiss published his famous treatise on the use of hypochlorite solution for the prevention of puerperal sepsis. Joseph Lister placed antisepsis on an even firmer footing with his work on the use of carbolic acid to counteract infection in compound fractures and thus encourage healing.

The discovery of antibiotics has undoubtedly been one of the greatest advances of the twentieth century and they have an important part to play in the treatment and prophylaxis of wound infections in the presence of spreading cellulitis.

Dressings have been produced, throughout the history of development of wound management from materials which were readily available and were rarely purpose designed to aid healing. Before 1870 there was probably no rational use of dressing materials but a variety were at the clinicians' disposal among them lint, charpie, oakum and cotton wool. Joseph Gamgee reported the use of his dressing pads in 1880 which were made of very soft cotton wool, rendered absorbent by the removal of oily matter, enveloped in bleached gauze. Gamgee pads have remained unchanged for almost a century.

During, and immediately after, the first World War attention was directed to the development of a non-adherent dressing. Tulle gras, was developed by the French Surgeon Lumiere and was prepared from cotton net which was impregnated with a mixture of soft paraffin and balsam of Peru. Since then various medicaments including penicillin and sulphonamides have been added.

For dressing chronic open ulcers the principle of a two-stage dressing developed consisting of two layers: the first providing a layer in contact with the wound surface, permeable to exudate and a second superimposed layer which absorbs the exudate which passes through the first layer. For the treatment of leg ulcers (particularly post-phlebitic ulcers) a variety of gauze dressings impregnated with zinc oxide or a long list of antiseptic agents became popular, their main purpose being for their antibacterial properties (the addition of zinc was for its relief of eczema and a theoretical advantage in healing).

### PATHOPHYSIOLOGY OF HEALING BY SECONDARY INTENTION

In open skin wounds, healing by secondary intention, the defect involves either the epidermis alone or, more usually, the epidermis and dermis combined. In epidermal defects restoration of continuity takes place without the formation of scar tissue but deeper defects restoration follows a non-specific process which leads to the deposition of scar tissue. The normal water-retaining properties of skin are lost in open wounds which leads to dehydration. The inflammatory exudate on the surface dries to form a crust, or scab, which may act as a barrier to infection from an external source but is permeable to water and allows the wound to dehydrate. This has a deleterious effect, however, because the epithelium which grows in from the sides of the wound needs to burrow deeply under the adherent crust to reach forming granulation tissue.

In 1962 George Winter reported that a wound which is kept moist, by an appropriate dressing, will epithelialise faster than a wound which has been allowed to form a crust. Hinman and Maibach confirmed this in 1963 on human volunteers. The concept of an 'ideal moist micro-environment' for healing was developed from this report and research since then has been aimed at producing new dressing materials which purport to serve this function. For example, if a wound is covered with one of the early dressing materials, such as gauze, then healing occurs as it would under the scab but if the wound is kept moist by covering it with a water-impermeable material, such as polyethylene film, then the epithelial cells migrate rapidly through the moist exudate on the surface of the wound.

Experimental studies have shown that increased oxygen tension in the wound atmosphere increases the oxygen tension in the wound itself, which further enhances re-epithelialisation and formation of granulation tissue. A rich blood supply to the wound is essential for rapid healing but all too often chronic ulcers, in man, suffer from a deficiency in blood supply (which is the case in arterial insufficiency and diabetic ulcers). In venous ulcers a possible cause of delayed healing is the deposition of fibrin around the skin capillaries causing a diffusion block and making oxygen less available to healing tissues.

### THE CONCEPT OF THE IDEAL DRESSING

Turner in 1979 identified characteristics that a dressing ought to have, if it were to provide the optimum micro-environment for healing to occur. These are as follows:

(1) Remove excess exudate and toxic components
(2) Maintain a high humidity at the dressing/wound interface
(3) Allow gaseous exchange
(4) Prevent heat loss
(5) Prevent secondary bacterial contamination
(6) Be free of toxic materials
(7) Allow painless re-dressings without causing trauma to the new granulation tissue.

To these must be added the important factor of cost.

The major difficulty of wound dressing research is the attempt to produce a dressing which will fulfil all these criteria. This accounts for the large number of different dressings that have appeared on the market over the past 10–15 years. In general these materials fulfil one or more parameters to a greater or lesser extent but so far a dressing has not appeared that fulfils all functions. Some of the potentially promising newer ones are briefly reviewed here.

Op-site (Smith and Nephew) was introduced as an incise drape but is now widely used as a dressing material, particularly over primarily sutured wounds. It is impermeable to water and micro-organisms but does allow gas and water vapour exchange. However, if a wound is infected and discharging large amounts of exudate then it may retain toxic substances which remain in contact with the wound surface thereby adversely affecting healing. Tegederm (Health Care Products) is very similar in structure and function.

Synthaderm (Armour Pharmaceutical Company) is a modified polyoxyethylene glycol which consists of a hydrophilic layer for contact with the wound surface and external hydrophobic layers with a 'closed' foam core. The occlusive nature of the contact surface is sufficient to maintain a moist wound interface whilst allowing gaseous exchange and excluding micro-organisms.

Lyophilised porcine skin epidermis (Johnson and Johnson) is a biological dressing which became available some ten years ago. It consists of a single layer of dermal collagen of 0.3 mm thickness and needs to be reconstituted as a soft pliable dressing by soaking in normal saline or Ringer's solution. It can enhance healing but the exact mode of action is far from clear.

Debrisan (Pharmacia) is a high molecular weight dextran polymer related to the polymers used in column chromatography. It is used primarily as a desloughing agent and is presented in fine bead form (0.1–0.3 mm). This dressing may absorb up to four times its own weight of exudate, simultaneously drawing bacteria from the wound into the interstices between the beads.

Silastic foam dressing (Dow Corning) is a silicone polymer foam which is formed by mixing a monomer solution with a catalyst. The mixture can be poured into an open wound, expands to four times the original volume, and sets in 3-4 minutes producing a perfect sponge cast. It is particularly suitable for the management of large or deep open wounds and sinuses and allows healing from the base upwards preventing skin bridges forming with resultant abscesses. The cast or stent is removed twice daily and may be soaked in a weak antiseptic solution prior to being squeezed dry and reinserted into the wound. As the wound closes new stents are periodically made to conform to the changing shape.

Lyofoam (Ultra) is a polyurethane, produced in sheets, which may be cut to size to suit any superficial wound or ulcer. It has a soft porous surface, backed by a larger celled foam, which enhances re-epithelialisation of a raw surface.

The most recent dressings to appear on this large market include a group known collectively as hydrogels. There are one or two dressings currently available: polyacrylamide (Geliperm-Geistlich) and polyethylene oxide (Vigilon-Bard). The former is a modification of a gel which is used in column chromatography and is produced as a strong sheet which provides a smooth moist surface to the wound with the capacity to absorb fluid. Vigilon is a softer material backed by a polyethylene sheet which also has absorptive capacity.

Several other products based on the hydrogel principle act also as debriding agents, among which are a dry form of polyacrylamide (Geistlich), a co-polymer starch hydrogel which comes in powder form (Bard), a graft T starch co-polymer (Schering) and gels which have other substances incorporated. Iodosorb (Stuart) contains iodine, Variclens (Dermal Laboratories) incorporates brilliant green.

This short review of the newer dressings makes no claim to be comprehensive. The search for an ideal wound dressing will doubtless continue but the aim of producing a 'universal dressing' may be as elusive as the 'Antidotarum universalis' of medieval times. It is likely that no single dressing will ever provide the best environment for all wounds because of the differing aetiologies and stages of healing and infection that exist in individual wounds. Random controlled clinical trials of the new dressings are almost impossible to execute successfully for this reason. Even the patient with similar bilateral venous ulcers cannot serve as his or her own control, as different rates of healing may be observed with identical treatments. Nevertheless attention to the optimal properties of dressings can only help increase their efficacy in new developments and hopefully increase cost-effectiveness which is increasingly a requirement of our NHS resources.

**Bibliography**

1. Majno, G. (1975) The Healing Hand; Man and Wound in the Ancient World, Cambridge, Massachusettes, Harvard University Press.
2. Forrest, R. D. (1982) Early history of wound treatment. *J. R. Soc. Med.,* **75** (3),198–205.
3. Forrest. R. D. (1982) Development of wound therapy from the dark ages to the present. *J. R. Soc. Med.,* **75** (4), 268–273.
4. Wangensteen, O. H., Wangensteen, S. D. (1978) The Rise of Surgery, Minneapolis, University of Minnesota Press.
5. Lister, J. (1867) 'On the antiseptic principle in the practice of surgery'. *Br. Med. J.,* **ii**, 246–248.
6. Bishop, W. J. (1959) A History of Surgical Dressings, Chesterfield, Robinson & Sons Ltd.
7. Winter, G. D., Scales, J. T. (1963) Effect of air drying and dressings on the surface of a wound. *Nature,* **197**, 91.
8. Hinman, C. D., Maibach, H. (1963) Effect of air exposure and occlusion on experimental human skin wounds. *Nature,* **200**, 377.
9. Turner, T. D. (1979) *The Pharmaceutical Journal,* **222**, 421.

